# Flexible and Stable Value Coding Areas in Caudate Head and Tail Receive Anatomically Distinct Cortical and Subcortical Inputs

**DOI:** 10.3389/fnana.2017.00106

**Published:** 2017-11-24

**Authors:** Whitney S. Griggs, Hyoung F. Kim, Ali Ghazizadeh, M. Gabriela Costello, Kathryn M. Wall, Okihide Hikosaka

**Affiliations:** ^1^Laboratory of Sensorimotor Research, National Eye Institute, National Institutes of Health, Bethesda, MD, United States; ^2^Department of Biomedical Engineering, Sungkyunkwan University (SKKU), Suwon, South Korea; ^3^Center for Neuroscience Imaging Research, Institute for Basic Science (IBS), Suwon, South Korea; ^4^National Institute on Drug Abuse, National Institutes of Health, Baltimore, MD, United States

**Keywords:** basal ganglia, caudate, neural pathways, object value learning, rhesus monkey

## Abstract

Anatomically distinct areas within the basal ganglia encode flexible- and stable-value memories for visual objects (Hikosaka et al., 2014), but an important question remains: do they receive inputs from the same or different brain areas or neurons? To answer this question, we first located flexible and stable value-coding areas in the caudate head (CDh) and caudate tail (CDt) of two rhesus macaque monkeys, and then injected different retrograde tracers into these areas of each monkey. We found that CDh and CDt received different inputs from several cortical and subcortical areas including temporal cortex, prefrontal cortex, cingulate cortex, amygdala, claustrum and thalamus. Superior temporal cortex and inferior temporal cortex projected to both CDh and CDt, with more CDt-projecting than CDh-projecting neurons. In superior temporal cortex and dorsal inferior temporal cortex, layers 3 and 5 projected to CDh while layers 3 and 6 projected to CDt. Prefrontal and cingulate cortex projected mostly to CDh bilaterally, less to CDt unilaterally. A cluster of neurons in the basolateral amygdala projected to CDt. Rostral-dorsal claustrum projected to CDh while caudal-ventral claustrum projected to CDt. Within the thalamus, different nuclei projected to either CDh or CDt. The medial centromedian nucleus and lateral parafascicular nucleus projected to CDt while the medial parafascicular nucleus projected to CDh. The inferior pulvinar and lateral dorsal nuclei projected to CDt. The ventral anterior and medial dorsal nuclei projected to CDh. We found little evidence of neurons projecting to both CDh and CDt across the brain. These data suggest that CDh and CDt can control separate functions using anatomically separate circuits. Understanding the roles of these striatal projections will be important for understanding how value memories are created and stored.

## Introduction

The caudate nucleus (CD) is involved in a variety of behaviors including voluntary movements, procedural learning and automatic behavior (skill or habit; Jog et al., 1999; Miyachi et al., 2002; Samejima et al., 2005; Ding and Gold, 2012; Hikosaka et al., 2017). Such different behaviors may be controlled by different regions of the CD (O’Doherty et al., 2004; Kim and Hikosaka, 2013; Yamamoto et al., 2013). Our previous data showed that two parallel pathways through the CD in the monkey contribute to reward-based learning, but in different ways (Kim and Hikosaka, 2013): the caudate head (CDh) encodes flexible values of visual objects based on short-term memories, while the caudate tail (CDt) encodes stable values of visual objects based on long-term memories. This raises an important question: are the short-term and long-term memories stored in CDh and CDt respectively, or in other brains areas that project to CDh and CDt selectively? As the first step to address this question, we need to know which brain areas project to CDh and CDt.

Many anatomical studies, particularly in monkeys, demonstrated that many cortical areas project to various regions of the striatum, including the CD (Kemp and Powell, 1970; Künzle, 1977, 1978; Yeterian and Van Hoesen, 1978; Selemon and Goldman-Rakic, 1985; Arikuni and Kubota, 1986; Saint-Cyr et al., 1990; Parthasarathy et al., 1992; Yeterian and Pandya, 1993; Cheng et al., 1997; Ferry et al., 2000). One of the first studies revealed that the CD receives topographical projections from the closest cortical regions, i.e., the frontal cortex (FC) projects to head, the parietal cortex (PC) projects to the body, and the temporal and occipital cortices project to the tail (Kemp and Powell, 1970). This model was later updated to show that multiple cortical areas project to the caudate in a topographic manner while other cortical regions send diffuse, non-topographic projections to the caudate (Selemon and Goldman-Rakic, 1985; Saint-Cyr et al., 1990). These studies, together, suggest that CDh and CDt receive inputs largely from different cortical areas, but partly from the same cortical areas. However, this conclusion is not sufficient to answer our specific question about neural circuit mechanisms: do CDh and CDt receive the same or different inputs from the cerebral cortex? This raises at least two anatomical questions. Do individual cortical neurons project to both CDh and CDt? Do projections to CDh and CDt arise from anatomically intermingled or distinct subpopulations of cortical neurons?

The CD is also known to receive inputs from many subcortical areas. Previous retrograde (Parent et al., 1983; Arikuni and Kubota, 1985; Jayaraman, 1985; Smith and Parent, 1986; Nakano et al., 1990; Saint-Cyr et al., 1990) and anterograde tracer studies (Russchen et al., 1985; Sadikot et al., 1992; McFarland and Haber, 2001) found that subcortical regions, including the thalamus, subthalamic nucleus (STN), amygdala, substantia nigra pars compacta (SNc) and dorsal raphe nucleus (DRN), project to the caudate. However, it is unclear whether different regions of the CD receive inputs from the same or different neurons in these subcortical areas. We recently obtained one answer to this question, specifically about the dopaminergic input to CDh and CDt (Kim et al., 2014). Dopamine neurons in the caudal-dorsal-lateral region of the SNc projected to CDt, while dopamine neurons in rostral-ventral-medial SNc projected to CDh. After finding this anatomical segregation of inputs from the SNc, we were curious whether other subcortical areas had similar segregation of their inputs to the CD. Here, we address the same two questions to other subcortical regions. Do individual subcortical neurons project to both CDh and CDt? Do projections to CDh and CDt arise from anatomically intermingled or distinct subpopulations of subcortical neurons?

To answer these questions, we injected different retrograde tracers into CDh and CDt so that we could identify three groups of neurons: neurons projecting only to CDh, neurons projecting only to CDt and neurons projecting both to CDh and CDt. We also examined the anatomical locations of these groups of neurons, especially when they were located close to each other. We found that CDh-projecting and CDt-projecting neurons were largely separate groups of neurons in both cortical and subcortical areas, although they were sometimes located close to each other.

## Materials and Methods

### General Procedures

The monkeys and most of the methods used in this article are the same as in previous studies (Kim and Hikosaka, 2013; Kim et al., 2014, 2017), but we present overall anatomical input data not published previously. All animal care and experimental procedures were approved by the National Eye Institute Animal Care and Use Committee and followed the Public Health Service Policy on the Humane Care and Use of Laboratory Animals. Briefly, we injected different retrograde tracers into functionally identified regions of the CDh and CDt in two monkeys (*Macaca mulatta*, male, 8–10 kg; monkeys ZO and SM). We chose the injection sites based upon single unit recordings where we identified the locations within CDh and CDt that respectively had the strongest short-term, flexible value responses or long-term, stable value responses. For detailed methods and injection sites, see these previous studies (Kim and Hikosaka, 2013; Kim et al., 2014).

### Injection

We injected cholera toxin subunit B conjugated to Alexa Fluor 555 (CTB555; 1% in 0.01 M PBS, pH 7.4; Thermo Fisher) into the CDt and Diamidino Yellow (DY; 2% in 0.2 M PBS, pH 7.2; Sigma) into the CDh of both monkeys. For the CDt injection, we used an injectrode comprised of a tungsten microelectrode (FHC) attached to a silica tube (outer/inner diameter: 155/75 μm; Polymicro Technologies). This allowed us to identify the dorsal and ventral borders of the CDt and inject the tracer into the middle of the volume, thus minimizing tracer leakage. For the CDh injection, we used a 30-gauge stainless-steel needle. After each injection, we waited 1 h before retracting the needle or injectrode from the injection site to minimize tracer backflow along the injection track. The summary of these injections and their locations can be seen in Table 1. The detailed injection method is described in a previous study (Kim et al., 2014).

**Table 1:** Summary of retrograde tracers used and injection site locations.

Monkey	CDh injection (short-term flexible value coding)	CDt injection (long-term stable value-coding)
ZO	0.6 μL DY; +3 mm of AC	0.3 μL CTB555; +14 mm of AC
SM	1.0 μL DY per injection site; +3 and 4 mm of AC	0.3 μL CTB555 per injection site; +13 and +14 mm of AC

*The different retrograde tracers used for caudate head (CDh) and caudate tail (CDt) injections in each monkey and their location relative to the anterior commissure*.

### Histology

After waiting 2 weeks for the tracers to be retrogradely transported, we deeply anesthetized monkeys SM and ZO and perfused with saline and 4% paraformaldehyde. After post-fixation and freezing the brains, we cut the brain into 50 μm sections using a sliding microtome. We collected every 250 μm-interval tissue section for plotting the fluorescently-labeled neurons and the adjacent tissue section for Nissl staining. The detailed histology method is described in a previous study (Kim et al., 2014).

### Topographical Analysis

We plotted the locations of fluorescently labeled neurons in each coronal section using a microscope stage digitizer (AccuStage) that allowed us to plot the locations of neurons with respect to the tissue section outline. After plotting the position of each neuron, we aligned the fluorescent neuron plots to the adjacent Nissl-stained section. For visualization purposes, we used Puppet Warp in Photoshop to adjust the fluorescent section to match the Nissl section and reduce anatomical distortions from mounting the tissue onto slides. We used two standard monkey atlases to identify and classify brain regions. To identify the anterior-posterior plane (relative to interaural line) and classify cortical areas, we used (Saleem and Logothetis, 2012). For subcortical areas, we used (Paxinos et al., 2008). For analyses looking at layer differences, we used immunofluorescent techniques to label cytoarchitecture on the same tissue section with plotted neurons to avoid any biases or errors potentially introduced by the nonlinear Puppet Warp. To calculate percentage of neurons double-labeled, we divided the number of identified double-labeled neurons across all sections by the total number of labeled neurons (single-labeled and double-labeled) plotted across all sections.

### Immunofluorescence and Immunohistochemistry

To examine which cortical layers projected to the CDh and CDt, we reacted six sections with anti-Neurofilament H antibodies (SMI-32; BioLegend Cat# 801701 RRID: AB_2564642). After permeabilizing the sections for 1 h (0.25% Triton X-100 in 0.05 M TBS), we blocked the sections for 1 h using a TBS solution containing 3% normal goat serum, 2% bovine serum albumin and 0.3% Triton X-100. The sections were then incubated overnight at room temperature with mouse monoclonal SMI-32 antibody (1:1000). We then washed the sections in PBS (3 × 10 min) and incubated the sections for 2 h at room temperature in goat anti-mouse IgG antibody conjugated with Alexa Fluor 647 (1:200, Thermo Fisher). Following three washes in PBS, the sections were air-dried overnight at room temperature and mounted with VECTASHIELD (Vector). Images were acquired using fluorescent and brightfield microscopes (Zeiss AXIO Imager M2 and Keyence BZ-X700). We adjusted the contrast and brightness of each color channel in Photoshop (Adobe) and ImageJ (NIH) to enhance the ability to differentiate fluorescently labeled neurons. This antibody has been shown to stain a subpopulation of pyramidal neurons in the neocortex (Campbell and Morrison, 1989; Hof and Morrison, 1995; Hof et al., 1996) and the pattern of labeling seen in this study matches well-established patterns from previous studies (Saleem et al., 2007; Ding and Van Hoesen, 2010).

## Results

### Value-Coding Properties of Injection Sites

**Figure 1:**
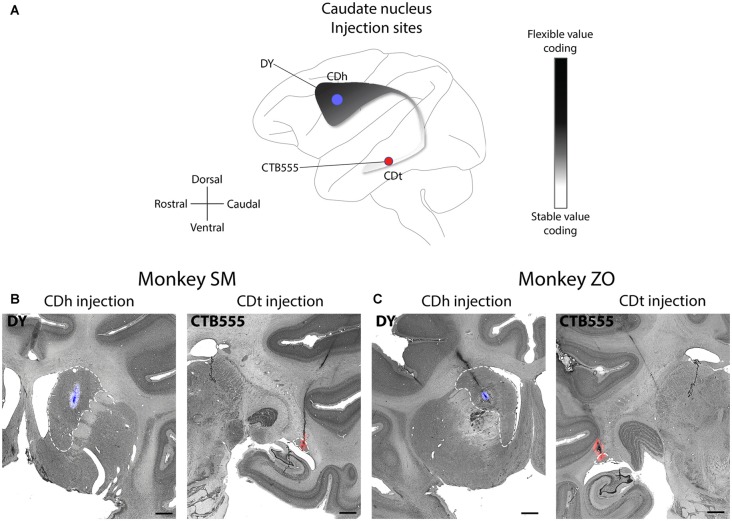
Functional and anatomical locations of caudate head (CDh) and caudate tail (CDt) injections. **(A)** Sagittal view of caudate nucleus (CD) showing stable-flexible value-coding gradient and the approximate location of the injections in both monkeys. **(B)** Nissl-stained coronal sections with fluorescent micrograph of injection sites overlaid for monkey SM. Dashed white line marks border of CD. Scale bars: 2 mm. **(C)** Same format as in **(B)**, but for monkey ZO.

Our underlying question was which brain regions contributed to the processing of flexible and stable values within the CDh and CDt. To help answer this question, we examined the anatomical inputs into distinct parts of the caudate by injecting retrograde neuronal tracers into CDh and CDt of two monkeys. We determined the injection sites by recording from different sites within CDh and CDt to find the particular areas within the CD where visually-responsive neurons encoded flexible value or stable value of visual objects. This was critical because object value-coding areas were localized in the caudate, especially within CDh. After mapping these locations, we injected retrograde tracers (Table 1) into the areas of the CDh and CDt with the most neurons encoding flexible- and stable-value, respectively (Figure 1A). See our previous article (Kim et al., 2014) for details on the localization of the injection sites to the portions of the CDh and CDt that respectively encoded flexible and stable values. For the CDt injections, we used injectrodes to precisely place the injection within the narrow volume of CDt. The injectrode allowed us to functionally locate stable-value coding neurons on the day of the injection and adjust the depth of the injectrode such that the tracer was injected at the same depth as the stable-value coding neurons. For the CDh, we used a 30-gauge injection needle aimed at the central CDh where we had previously found the highest density of flexible-coding neurons. The injection sites are shown for monkey SM (Figure 1B) and ZO (Figure 1C).

### Brain-Wide Distribution of CDh- and CDt-Projecting Neurons

Many brain regions contained exclusively DY-labeled neurons (CDh-projecting) or exclusively CTB555-labeled neurons (CDt-projecting). The remaining regions contained a mixture of both CDh-projecting and CDt-projecting neurons, but the CDh-projecting and CDt-projecting neurons were often spatially segregated rather than intermingled. Less than 1% of all labeled neurons were double-labeled with DY and CTB555 tracers, indicating that CDh and CDt receive inputs almost exclusively from different neurons. Due to the low number of double-labeled neurons found, we could not identify any pattern to the distribution of double-labeled neurons. Dopaminergic inputs to CDh and CDt were described in previous study (Kim et al., 2014). We here examined overall brain inputs, except the dopaminergic inputs, to CDh and CDt.

**Figure 2:**
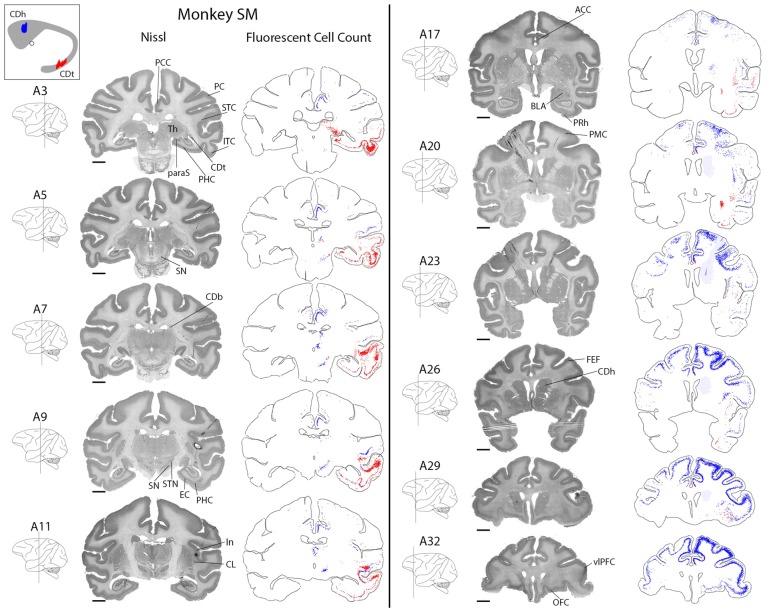
Overview of CDh and CDt projections in monkey SM. Nissl-stained coronal sections juxtaposed along fluorescent section outlines with plotted CDh-projecting (blue; DY-labeled) and CDt-projecting (red; CTB555-labeled) neurons. Shaded areas in fluorescent sections show regions with increased background fluorescence from tracer leakage. Colored areas of caudate nucleus inset show extent of CDt (red) and CDh (blue) injection sites. Fifty micrometer between juxtaposed Nissl-stained and fluorescent sections. Anterior-posterior position relative to the interaural line. Scale bar: 5 mm.

**Figure 3:**
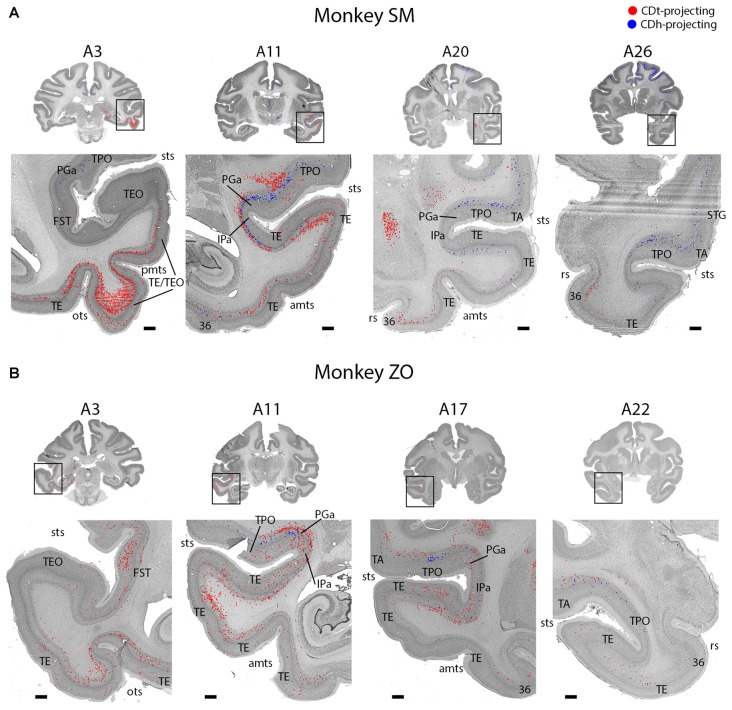
Topographic projections from temporal cortex to CDh and CDt. **(A)** Overview coronal sections showing CDh (blue; DY-labeled neurons) and CDt (red; CTB555-labeled neurons) projecting neurons in monkey SM. Boxed regions of overview sections shows magnified region of temporal cortex. Plotted neurons from fluorescent sections overlaid onto Nissl-stained sections. Nissl-stained sections 50 μm anterior to fluorescent sections with plotted neurons. Anterior-posterior position relative to interaural line. Scale bars: 1 mm. **(B)** Example coronal sections from monkey ZO. Same format as **(A)**. Abbreviations: sts, superior temporal sulcus; pmts, posterior middle temporal sulcus; ots, occipitotemporal sulcus; rs, rhinal sulcus; amts, anterior middle temporal sulcus.

**Figure 4:**
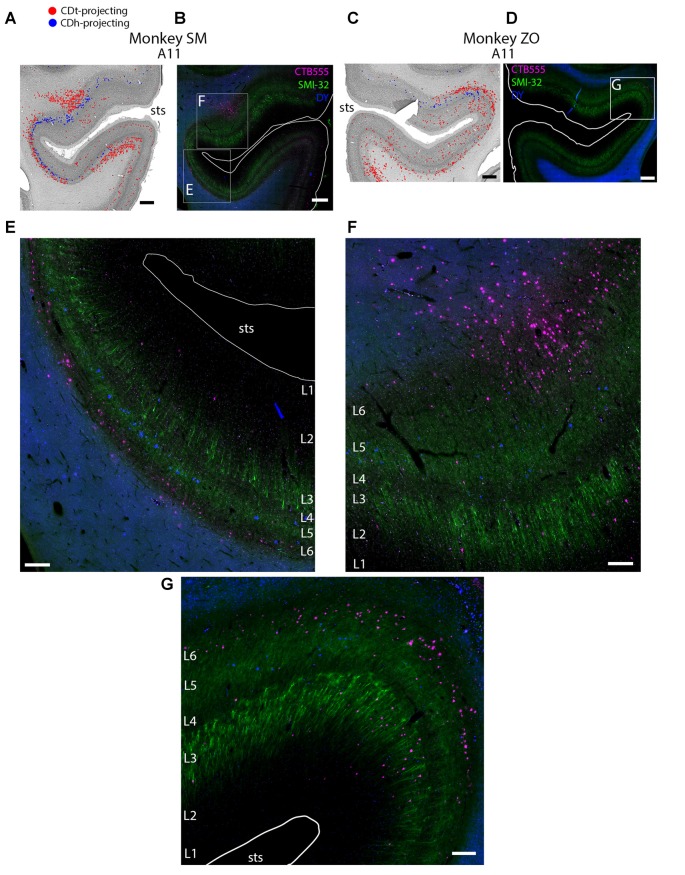
Different layers within STS cortex projected to CDh and CDt. **(A)** Coronal section showing CDh- and CDt- projecting neurons in STS cortex of monkey SM. Same format as Figure 3. **(B)** Fluorescent image of STS region showing CDh-projecting neurons (blue), CDt-projecting neurons (magenta) and cell layers (green; SMI32-positive). Fifty micrometer anterior to Nissl-stained section in **(A)**. Boxed regions examined in **(E,F)**. White line shows section border. Scale bar: 1 mm. **(C)** Coronal section showing STS cortex of monkey ZO. Same format as **(A)**. **(D)** Fluorescent image of STS region in monkey ZO. Same format as **(B)**. Boxed region examined in **(G)**. **(E)** CDt-projecting neurons (magenta) are in Layer 6. CDh-projecting neurons (blue) are in Layer 3 and 5. Scale bar: 250 μm. **(F)** CDt-projecting neurons are in Layer 3 and 6 while CDh-projecting neurons are in Layer 5. Same format as **(E)**. **(G)** CDt-projecting neurons (magenta) are in Layers 3 and 6. CDh-projection neurons (blue) are in Layer 5. Same format as **(E)**. Abbreviations: sts, superior temporal sulcus.

**Figure 5:**
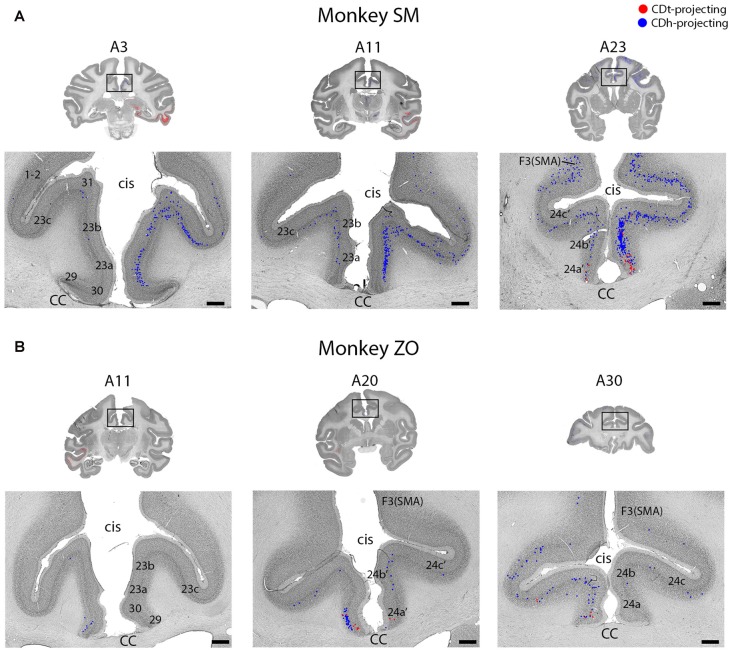
Intermingled projections from cingulate cortex to CDh and CDt. **(A)** Coronal sections showing intermingled CDh and CDt projecting neurons in cingulate cortex of monkey SM. Same format as Figure 3. **(B)** Coronal sections showing intermingled CDh and CDt projecting neurons in cingulate cortex of monkey ZO. Same format as Figure 3. Abbreviations: cis, cingulate sulcus; CC, corpus callosum; SMA, supplementary motor area.

**Figure 6:**
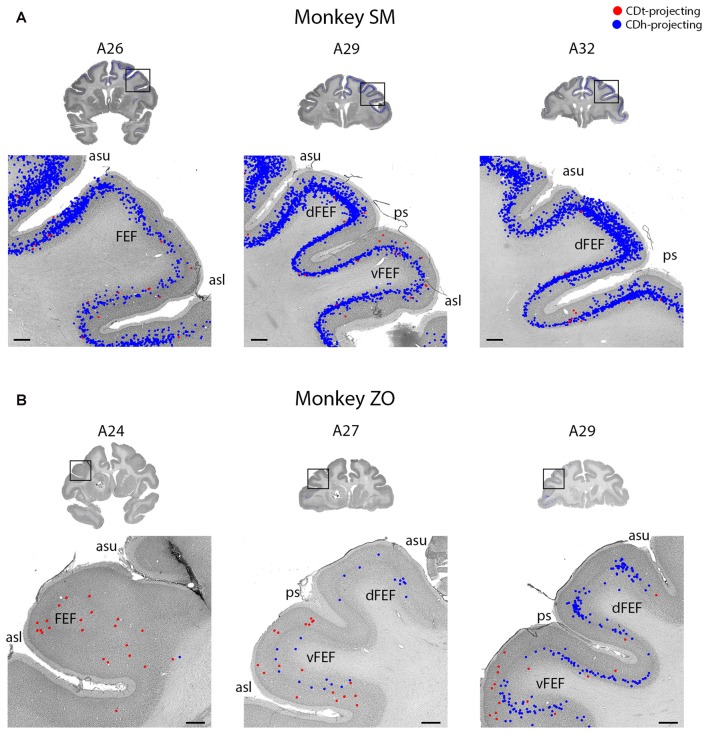
FEF projected to CDh and CDt. **(A)** Coronal sections from monkey SM showing CDh- and CDt-projecting neurons in FEF. Same format as Figure 3. **(B)** Coronal sections from monkey ZO showing CDh- and CDt-projecting neurons in FEF. Same format as Figure 3. Abbreviations: asu, upper limb of arcuate sulcus; asl, lower limb of arcuate sulcus; ps, principal sulcus; FEF, frontal eye field.

**Figure 7:**
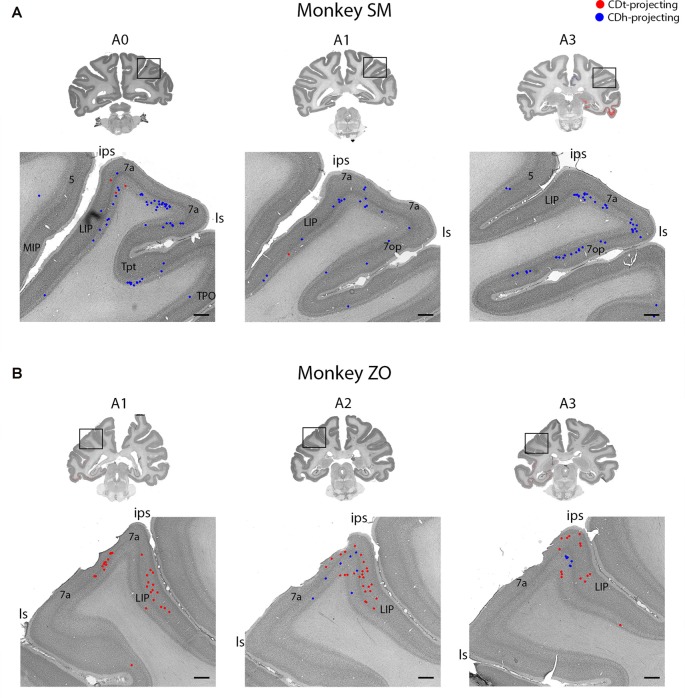
Parietal cortex (PC) contained neurons projecting to CDh and CDt. **(A)** Coronal sections from monkey SM showing CDh- and CDt-projecting neurons in PC. Same format as Figure 3. **(B)** Coronal sections from monkey ZO showing CDh- and CDt-projecting neurons in PC. Same format as Figure 3. Abbreviations: ips, intraparietal sulcus; ls, lateral sulcus; LIP, lateral intraparietal area; MIP, medial intraparietal area.

**Figure 8:**
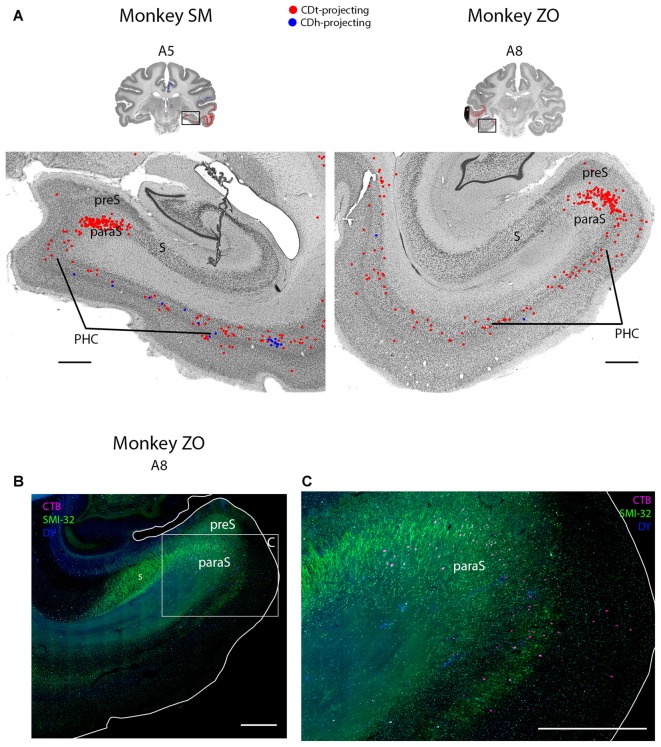
PHC and parasubiculum project to CDt. **(A)** Coronal sections from monkeys SM and ZO showing CDh- and CDt-projecting neurons in PHC and parasubiculum. Same format as Figure 3. **(B)** Fluorescent section showing CDt-projecting neurons (red) in parasubiculum of monkey ZO. SMI32-labeling used to identify subiculum, presubiculum, parasubiculum and PHC. Boxed region examined in **(C)**. Scale bar: 1 mm. **(C)** CDt-projecting neurons are confined to parasubiculum and layer 3 and 5 of PHC. Same format as **(B)**. Scale bar: 1 mm. Abbreviations: PHC, parahippocampal cortex; S, subiculum; preS, presubiculum; paraS, parasubiculum.

**Figure 9:**
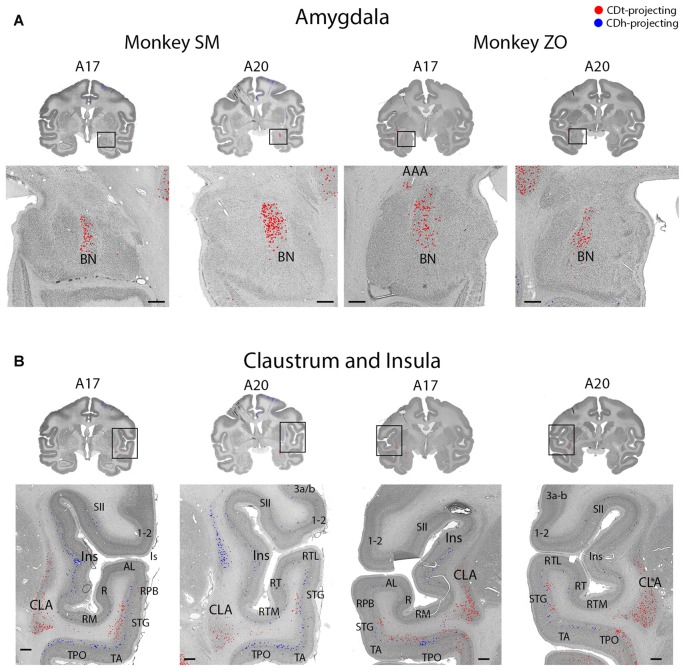
Topographic projections from amygdala and claustrum to CDh and CDt. **(A)** Coronal sections showing CDt-projecting neurons in dorsal basal nucleus of amygdala. Same format as Figure 3. **(B)** Coronal sections showing CDt-projecting neurons in caudal-ventral claustrum and CDh-projecting neurons in rostral-dorsal claustrum. Same format as Figure 3. Abbreviations: BN, basal nucleus; AAA, anterior amygdaloid area; CLA, claustrum; Ins, insula; ls, lateral sulcus; sts, superior temporal sulcus.

**Figure 10:**
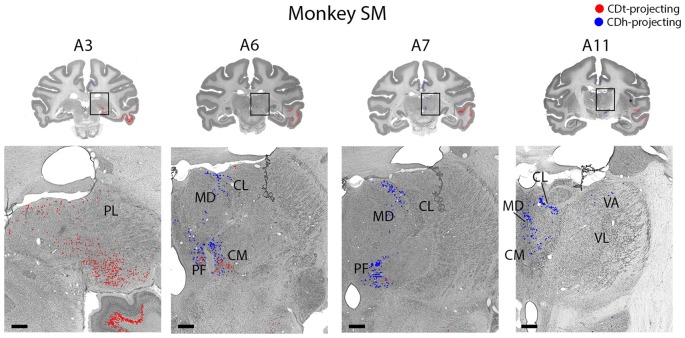
Topographic projections from thalamic nuclei to CDh and CDt. Coronal sections from monkey SM showing CDh- and CDt-projecting neurons in pulvinar, centromedian, parafascicular, ventral anterior and mediodorsal thalamic nuclei. Same format as Figure 3. Abbreviations: PF, parafascicular nucleus; CM, centromedian nucleus; MD, medial dorsal nucleus; CL, central lateral nucleus; PL, pulvinar; LD, lateral dorsal nucleus; VA, ventral anterior nucleus; VL, ventral lateral nucleus.

**Figure 11:**
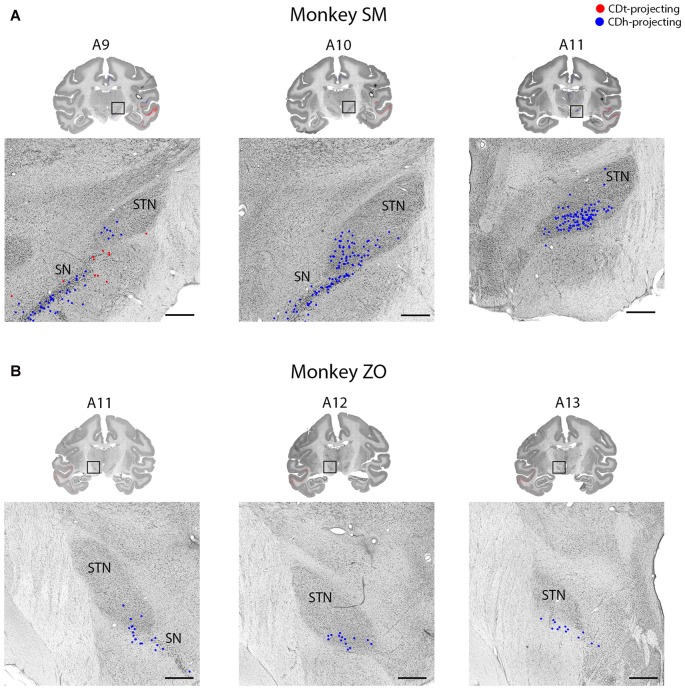
STN projects to CDh. **(A)** Coronal sections from monkey SM showing CDh-projecting neurons in ventral STN. Same format as Figure 3. **(B)** Coronal sections from monkey ZO showing CDh-projecting neurons in ventral STN. Same format as Figure 3. Abbreviations: SN, substantia nigra; STN, subthalamic nucleus.

CDh received cortical inputs mainly from the anterior regions of the brain (Figure 2, blue), especially from prefrontal (PFC; A26–A32), orbitofrontal (OFC; A26–A32), anterior cingulate (ACC; A20–A32) and posterior cingulate cortex (PCC; A3–A11). These cortical inputs were bilateral, although ipsilateral inputs were more numerous. These regions contained the most CDh-projecting neurons, but we also found CDh-projecting neurons in premotor cortex (PMC; A20–A32), superior temporal cortex (STC; A5–A26), inferior temporal cortex (ITC; A9–A26), PC (A3–A7) and insular cortex (A11–A26). Across all cortical regions examined, we found CDh-projecting neurons in layer 5 and occasionally in layer 3. The cortical distribution of CDh-projecting neurons is shown in Figures 3–7. CDh also received inputs from multiple subcortical areas, including the rostral intralaminar thalamic nuclei (A5–A11) and rostral-dorsal claustrum (A20–A29). The subcortical distribution of CDh-projecting neurons is shown in Figures 8–11.

CDt received cortical inputs mainly from the posterior regions of the brain (Figure 2, red). Cortical inputs originated mainly from ITC (A3–A20) and STC (A7–A17), exclusively from the ipsilateral side. We also found CDt-projecting neurons in OFC, PFC (A26–A32) and ACC (A17–A32), but fewer neurons than in the aforementioned posterior regions. The cortical distribution of CDt-projecting neurons is shown in Figures 3–7. We found CDt-projecting neurons in layer 3 and 5, except for in the STC and specific regions of ITC. In STC regions and dorsal ITC (A5–A11), we found CDt-projecting neurons in layers 3 and 6 (not layer 5). In specific subregions of ventral ITC (A3), we found CDt-projecting neurons in layers 3, 5 and 6. These labeled neurons in different cortical layers are shown in Figures 3, 4.

**Figure 12:**
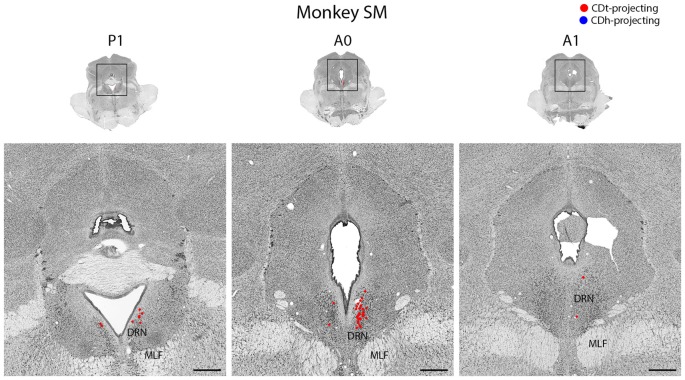
DRN projects to CDt. Coronal sections from monkey SM showing CDt-projecting neurons in DRN. Same format as Figure 3. Abbreviations: DRN, dorsal raphe nucleus; MLF, medial longitudinal fasciculus.

Robust inputs to CDt also originate from subcortical areas, predominately the inferior pulvinar (A3), caudal-lateral intralaminar thalamic nuclei (A5), amygdala (A17–A23), caudal-ventral claustrum (A17–A23), pre/para subiculum (A3–A7) and DRN (A0; not shown in Figure 2). These subcortical areas with CDt-projecting neurons are shown in Figures 8–12.

### Cortical Regions

#### Temporal Cortex Projects to Both CDt and CDh

STC and ITC contained both CDh- and CDt-projecting neurons (Figures 2, 3; A3–A26). We found many CDt-projecting neurons within ITC (A3–A11) and caudal-medial STC (A7–A11) and fewer CDh-projecting neurons within STC (A3–A26). Many of these regions in STC and ITC contained both CDh-projecting and CDt-projecting neurons. We also found sporadic clusters of CDh-projecting neurons within specific areas of ventral ITC (A7–A11). For CDt projecting neurons, we found a rostral-caudal topography in ITC. Rostral-dorsal ITC contained few CDt-projecting neurons (A17–A26). In the intermediate dorsal ITC, the number of CDt-projecting neurons increased (A9–A11). In the caudal ITC, the region with the highest density of CDt-projecting neurons shifted progressively more ventral until the last section analyzed (A3), where ventral ITC contained the most CDt-projecting neurons.

#### Different Layers of Temporal Cortex Contain CDh- and CDt-Projecting Neurons

These data show that CDh and CDt receive inputs from many of the same regions in the temporal cortex, although the ratio of neurons projecting to CDh vs. CDt varied. However, this may not indicate that the same neuron populations project to both CDh and CDt. In fact, we found that different neuron layers of STC and ITC contained CDh-projecting and CDt-projecting neurons, respectively (Figure 3). Throughout STC and dorsal ITC regions, CDt- and CDh-projecting neurons were located in separate layers with CDh-projecting neurons deeper than CDt-projecting neurons (Figures 3A, A11; 3B, A11–A22). Do these layers correspond to layer 5 and layer 6, respectively?

To address this question, we used an anti-Neurofilament H antibody (SMI-32) to demarcate the cortical layer boundaries. This antibody has previously been shown to primarily label layer 5 neurons within STC (Cusick et al., 1995). The SMI-32 data confirmed that layer 6 contained CDt-projecting neurons while layer 5 neurons contained CDh-projecting neurons (Figure 4). We also found that layer 3 contained some CDh-projecting or CDt-projecting neurons. Interestingly, within ventral ITC (Figure 3A, A3), layers 3, 5 and 6 contained CDt-projecting neurons. Notably, we did not find this separation of CDh-projecting and CDt-projecting neurons to different layers for other cortical regions, including ACC, PCC, PMC, PC, or PFC. In these other regions, we saw intermingled CDh-projecting and CDt-projecting neurons in layers 3 and 5, with more labeled neurons in layer 5.

#### Cingulate Cortex Projects Non-topographically to CDh and CDt

Layer 5 of cingulate cortex in both hemispheres contained intermingled CDh-projecting and CDt-projecting neurons (Figures 2, 5; A3–A30), with more labeled neurons in the ipsilateral hemisphere. We found CDt-projecting neurons in the medial-ventral ACC and found CDh-projecting neurons in both the ACC (Figure 2, A17–A26) and PCC (Figure 2, A3–A11). The rostral-medial ACC contained the most CDh-projecting neurons (Figures 5A,B, A23–A30). Although cingulate cortex contained more CDh-projecting neurons than CDt-projecting neurons, the CDh-projecting and CDt-projecting neurons were intermingled suggesting that the ACC regions projected non-topographically to both CDh and CDt.

#### Prefrontal and Frontal Cortex Project Primarily to CDh, with Fewer CDt-Projecting Neurons

The PFC and FC contained CDh-projecting and CDt-projecting neurons, with more CDh-projecting neurons (Figure 2, A23–A32). We found most labeled neurons within layer 5, with occasional labeled neurons in layer 3. Many of the dorsal PFC and FC regions (dorsal PMC and supplementary motor area) contained only CDh-projecting neurons. More ventral regions, including the ventral PMC, ventral-lateral PFC and dorsal-lateral PFC contained many CDh-projecting neurons with some CDt-projecting neurons intermingled. OFC also contained many CDh-projecting neurons with occasional CDt-projecting neurons intermingled. The frontal eye field (FEF) showed some weak topography (Figure 6). In the dorsal FEF, we found almost exclusively CDh-projecting neurons while we found CDh-projecting and CDt-projecting neurons within the ventral FEF. In addition, layer 3 of the caudal-ventral FEF contained many CDt-projecting neurons (in addition to CDt-projecting neurons in layer 5) while CDh-projecting neurons were primarily found in layer 5 with few neurons in layer 3.

#### Parietal Cortex Projects to CDh and CDt

Within the PC, several regions contained CDh-projecting neurons while fewer regions contained CDt-projecting neurons (Figure 2, A3–A7). Within the dorsal bank of the lateral sulcus, we found sporadic clusters of CDh-projecting neurons, but no neurons labeled from the CDt injection site. The dorsal part of the lateral intraparietal cortex (LIP) contained a few CDh-projecting or CDt-projecting neurons (Figure 7). There were differences between the two monkeys for LIP. In monkey ZO, LIP contained more CDt-projecting neurons than CDh-projecting neurons (Figure 7B) while in monkey SM, LIP showed the opposite pattern of more CDh-projecting neurons (Figure 7A).

#### Entorhinal, Perirhinal and Parahippocampal Cortex Contained Primarily CDt-Projecting Neurons with Occasional CDh-Projecting Neurons

Within the medial temporal lobe, many regions contained dense amounts of CDt-projecting neurons and occasional CDh-projecting neurons (Figures 2, A3–A26; 8A). The caudal perirhinal cortex contained many CDt-projecting neurons with no neurons labeled from the CDh injection site. We did not find any labeled neurons within rostral perirhinal cortex (Figure 2, A17–A26). The caudal entorhinal cortex (EC) contained many CDt-projecting neurons and occasional CDh-projecting neurons (Figure 2, A9–A11). We did not find any labeled neurons in more rostral regions of the EC (Figure 2, A17–A23). The parahippocampal cortex (PHC) contained many CDt-projecting neurons and occasional CDh-projecting neurons (Figures 2, A3–A11; 8A). Within the parasubiculum, we also found many CDt-projecting neurons, but no neurons labeled from the CDh injection site (Figure 8). To identify what region of the subiculum projected to CDt, we used the anti-Neurofilament H antibody (SMI-32) to label the cytoarchitecture within the parahippocampal and subiculum regions. The SMI-32 antibody has been previously used to identify subregions of the subiculum (Ding and Van Hoesen, 2010; Ding, 2013). This immunofluorescence confirmed that parasubiculum contained CDt-projecting neurons (Figures 8B,C). We did not find any labeled neurons in the presubiculum or subiculum.

#### Insular Cortex Projects Primarily to CDh with Occasional CDt Projections

Located between the parietal and temporal cortex, the dysgranular and granular insular cortex contained CDh-projecting and CDt-projecting neurons, with more CDh-projecting neurons (Figures 2, A7–A26; 9B). Rostral and caudal insula both contained scattered CDt-projecting neurons. We found CDh-projecting neurons within the rostral and caudal insula with the most neurons in the rostral insula.

### Subcortical Regions

#### Amygdala Projected to CDt

The ipsilateral amygdala contained many CDt-projecting neurons, but no neurons labeled from the CDh injection site (Figure 9A). The dorsal basolateral amygdala (BLA) contained the highest number of CDt-projecting neurons with more sporadic CDt-projecting neurons in the intermediate subdivision of the BLA. In monkey ZO, we also found CDt projecting neurons in the anterior amygdaloid area (AAA; Figure 9A, right A17). In monkey SM, we found no AAA neurons labeled from the CDt injection site. No region of the amygdala contained neurons labeled from the CDh injection sites in either monkey.

#### Claustrum Projected to CDh and CDt from Anatomically Distinct Subpopulations

The ipsilateral claustrum contained many CDh-projecting and CDt-projecting neurons, but in anatomically segregated subdivisions (Figure 9B). The CDh-projecting neurons were in the rostral tip of the claustrum and extended through the intermediate claustrum (A20). As the CDh-projecting neurons decreased in number and disappeared in the intermediate claustrum, CDt-projecting neurons began to appear in the ventral claustrum (A20). Although CDh- and CDt- projecting neurons were intermingled in the intermediate claustrum, we did not find any double-labeled neurons projecting to both CDh and CDt. In the caudal claustrum, the number of CDt-projecting neurons increased and extended dorsally (A17). Overall, our findings indicated a gradient to the claustrum projections. The rostral-dorsal claustrum contained CDh-projecting neurons while the caudal-ventral claustrum contained CDt-projecting neurons.

#### Thalamic Nuclei Connect Differently to CDh and CDt

Within the thalamus, several ipsilateral nuclei contained CDh-projecting and CDt-projecting neurons (Figure 10), but displayed varied patterns of labeled neuron distribution. The medial and inferior pulvinar contained CDt-projecting neurons, but no neurons labeled from the CDh injection site (Figure 10, A3). We found these CDt-projecting neurons primarily in the medial half of the pulvinar nucleus. The lateral mediodorsal nucleus (MD) and medial central lateral nucleus (CL) contained CDh-projecting neurons (Figure 10, A5–A7). The lateral dorsal nucleus (LD) contained CDt-projecting neurons, but no neurons labeled from the CDh injection site (Figure 10, A9). In monkey SM, we found a small number of CDh-projecting neurons within the dorsal VA nucleus (Figure 10, A11). In monkey ZO, we did not find any labeled neurons within the ventral anterior (VA) or ventral lateral (VL) nuclei of the thalamus.

The caudal intralaminar nuclei projected topographically to CDh and CDt in both monkeys (Figure 10, A5–A9). We found both CDh-projecting and CDt-projecting neurons within the centromedian-parafascicular complex (CM-PF), but within different subregions. CDh-projecting neurons were located more rostral-medial and generally confined to within the medial PF, while CDt-projecting neurons were located more caudal-lateral and were generally confined to within the lateral PF and medial CM. These results show a topographical projection pattern from the CM-PF complex to CDh and CDt with the medial PF containing primarily CDh-projecting neurons and the lateral PF and medial CM containing primarily CDt-projecting neurons.

#### Subthalamic Nucleus Projects to CDh

Within the STN of both monkeys, we found ipsilateral CDh-projecting neurons, but no neurons labeled from the CDh injection site (Figure 11). The highest number of CDh-projecting neurons was in the caudal third of the STN and concentrated within the ventromedial region of the STN.

#### Dorsal Raphe Nucleus Projected to CDt

In both monkeys, the DRN contained CDt-projecting neurons, but no neurons labeled from the CDh injection site (Figure 12). We found CDt-projecting neurons in both the ipsilateral and contralateral DRN, but the ipsilateral DRN contained more neurons. The CDt-projecting neurons were restricted to a narrow anterior-posterior range, with the CDt-projecting neurons found within 1 mm of the interaural coronal plane.

## Discussion

Our results support anatomically distinct pathways through CDh and CDt, which respectively encode flexible and stable values of visual objects. CDh and CDt received projections from largely separate cortical and subcortical regions with few neurons simultaneously projecting to both CDh and CDt. These results support previous anatomical studies that examined the cortical projections to either CDh or CDt, and further reveal novel results about the topography of projections to CDh and CDt.

### Cortical Projections

Overall, we found a topographic projection pattern from the cortex to the striatum, as described in previous studies (Johnson et al., 1968; Kemp and Powell, 1970; Goldman and Nauta, 1977; Yeterian and Van Hoesen, 1978; Selemon and Goldman-Rakic, 1985; Saint-Cyr et al., 1990; Cheng et al., 1997; Ferry et al., 2000). In this topographic pattern, anterior cortical regions contained more CDh-projecting neurons while posterior cortical regions contained more CDt-projecting neurons.

#### Inferior Temporal Cortex

ITC contained many CDt-projecting neurons, but fewer CDh-projecting neurons (Figure 3). This result agrees with earlier findings (Kemp and Powell, 1970; Saint-Cyr et al., 1990; Baizer et al., 1993; Steele and Weller, 1993; Webster et al., 1993; Cheng et al., 1997). The numerous CDt-projecting neurons in ITC may contribute to both the high capacity memory and accurate object recognition of CDt neurons (Yamamoto et al., 2013) due to the object-selectivity observed in ITC area TE (Tamura and Tanaka, 2001). Previous studies found that the visual responses of area TE neurons are modulated by recent object-reward association (Liu and Richmond, 2000; Mogami and Tanaka, 2006; Kaskan et al., 2017). However, our anatomical data suggest that ITC may encode long-term stable values of visual objects, which is supported by our recent fMRI study from our lab (Ghazizadeh et al., 2016).

#### Laminar Organization of Inputs to the Caudate Nucleus

CDh- and CDt-projecting neurons were located in different cortical layers of the STC and dorsal ITC (Figure 4). Layer 5 contained exclusively CDh-projecting neurons, layer 6 contained exclusively CDt-projecting neurons, and layer 3 contained intermingled CDh-projecting neurons and CDt-projecting neurons. Interestingly, while the cortical projections to striatum are commonly thought to only originate from layers 3 and 5 (Kitai et al., 1976; Jones et al., 1977; Oka, 1980; Royce, 1982; Arikuni and Kubota, 1986), recent evidence have also revealed layer 6 projections consistent with our finding. In rodents, layer 6 neurons project to the patch, not matrix, in the striatum (Gerfen, 1989; Kincaid and Wilson, 1996). In addition, layer 6 neurons of the mouse prelimbic cortex project to the ventromedial striatum (Hintiryan et al., 2016). In fact, a close examination of Saint-Cyr et al. (1990) reveals that within ITC, neurons in layer 6 (in addition to layers 3 and 5) project to CDt (see Figures 4B,D of Saint-Cyr et al., 1990).

Our data suggest that layers 5 and 6 of the STC and ITC send different information to CDh and CDt respectively. Possibly related to this idea, several articles have reported unique anatomy and functions for specific cortical layers (Fries and Distel, 1983; Iwamura et al., 1983, 1985; Fries, 1984; Tehovnik et al., 2002; Takeuchi et al., 2011; Olsen et al., 2012; Bortone et al., 2014; Koyano et al., 2016). Notably, some studies (Olsen et al., 2012; Bortone et al., 2014) suggest that different brain areas may regulate visual processing by sending convergent inputs to layer 6, thus inhibiting layers 2–5. A similar function might occur in the STC and ITC, such that the information sent to CDt (through layer 6) may inhibit the information sent to CDh (through layer 5).

#### Projection of Scene-Selective Areas to CDt

The parahippocampal and cortical areas near the occipitotemporal sulcus (areas TEpv, TFO, TF and TH) contained many CDt-projecting neurons (Figures 2, A3–A26; 8A), confirming an earlier study (Saint-Cyr et al., 1990). These cortical areas are sensitive to images of scenes (Kornblith et al., 2013), suggesting that CDt neurons may encode visual environment, in addition to visual objects.

#### Projection of Cingulate and Frontal Cortices to CDh

Many neurons in the ACC and PCC projected to ipsilateral and/or contralateral CDh (Figure 5). This agrees with previous anatomical studies (Kemp and Powell, 1970; Yeterian and Van Hoesen, 1978; Selemon and Goldman-Rakic, 1985; Saint-Cyr et al., 1990; Parvizi et al., 2006). Our data further showed that ACC and PCC send fewer inputs to CDt. ACC and PCC are known to contribute to flexible decision making (Pearson et al., 2011; Heilbronner and Hayden, 2016), while CDh processes object values flexibly (Kim and Hikosaka, 2013) These data suggest that the connections of ACC and PCC to CDh are key mechanisms of flexible decision making.

Similar mechanisms may be shared by the prefrontal and frontal cortices, which are shown to project to ipsilateral and/or contralateral CDh (Figures 2, 6) in agreement with previous studies (Parthasarathy et al., 1992; Ferry et al., 2000; Haber et al., 2006). Our data, however, showed that some ipsilateral neurons in these areas projected to CDt (Figures 2, A23–A32; 6). The connections of the frontal and cingulate cortices to CDt might contribute to stable decision making.

#### Moderate, Intermingled Projections from Parietal Cortex to CDh and CDt

Previous studies showed that the PC did project mostly to the dorsal part of the CDh (Kemp and Powell, 1970; Weber and Yin, 1984; Selemon and Goldman-Rakic, 1985, 1988; Cavada and Goldman-Rakic, 1991; Baizer et al., 1993; Yeterian and Pandya, 1993). Instead, we injected the tracer into the central part of CDh (Figure 1) where visual neurons showed flexible value coding. Possibly due to the localized tracer injection in CDh, there were relatively few retrograde-labeled neurons in the PC (Figure 7). The projection to CDt also was not prominent. These data may suggest that the PC is involved in behavior that is not simply related to object values.

### Subcortical Projections

#### Different Thalamic Nuclei Projected to Different Parts of Caudate

Within the thalamus, many of the nuclei projected in a topographic pattern to CDh or CDt. For example, CDh receives projections from the rostral-medial PF while CDt receives projections from the ventral-lateral PF and medial CM (Figure 10), supporting a recent study (Ito and Craig, 2008). A previous study reported that neurons in the CM-PF complex respond to salient events or stimuli, including unexpected, novel and rewarded objects (Matsumoto et al., 2001). These types of information might be integrated with the flexible or stable values encoded in CDh and CDt circuits.

The pulvinar contained many CDt-projecting neurons, but no neurons labeled from the CDh injection site. To the best of our knowledge, this is the first report of CDt projections from the pulvinar. A previous tracer study in squirrel monkey (Smith and Parent, 1986) showed projections from the medial pulvinar to CDh and putamen, but did not examine CDt. By comparing the results from their large CDh injection site and our smaller CDh injection site, this suggests that the pulvinar projects to a different part of CDh than the flexible-coding visual CDh of our injection site.

#### Topographic Projections from Claustrum to CDh and CDt

To our knowledge, this is the first report of CDt-projecting neurons within the claustrum. We found that CDh received projections from the dorsal-rostral claustrum, consistent with previous studies in monkeys (Arikuni and Kubota, 1985; Park et al., 2012) and humans (Milardi et al., 2015). In contrast, CDt received projections from the caudal-ventral claustrum. Functional studies have revealed that different parts of the claustrum respond to different sensory modalities. In primates, the central claustrum responds to auditory stimuli while the ventral claustrum responds to visual stimuli (Remedios et al., 2010). In cats, there are similar anatomical areas that respectively respond to auditory, somatosensory and visual stimuli (Olson and Graybiel, 1980). However, the functions of these claustrum sub-regions are unclear. Our finding that anatomically distinct territories of the claustrum respectively project to CDh and CDt may help identify possible functions for the claustrum in the future.

#### Basolateral Amygdala Projects to CDt

Within the amygdala, we found numerous CDt-projecting neurons in the BLA of both monkeys. We found no neurons labeled from the CDh injection site in any region of the amygdala. Our findings were similar to previous tracer studies in monkey (Parent et al., 1983; Arikuni and Kubota, 1985; Russchen et al., 1985; Saint-Cyr et al., 1990; Friedman et al., 2002; Fudge et al., 2002, 2004; Cho et al., 2013; deCampo and Fudge, 2013). Several tracer studies (Arikuni and Kubota, 1985; Russchen et al., 1985; Fudge et al., 2002) reported that some neurons in amygdala project to CDh, but these projections were to the ventral CDh rather than the central CDh where we injected our retrograde tracers. Since the amygdala is critical for the emotional effects on behavior (LeDoux, 2000; Pessoa and Adolphs, 2010), the robust projection from the amygdala to CDt suggests that emotional experiences contribute to the stable, rather than flexible, coding of reward values.

#### Subthalamic Nucleus Projects to CDh

Our results support the previous findings that reported topographical projections from the STN to the caudate and putamen (Parent and Smith, 1987; Nakano et al., 1990; Smith et al., 1990). In our study and these previous articles, neurons in the medial STN projected to CDh. A previous article from our lab (Isoda and Hikosaka, 2008) suggests a possible role for the STN in flexible decision making: STN neurons increased their firing specifically before controlled saccades switched from automatic saccades. The projection of STN to CDh may contribute to the automatic-to-controlled switching behavior.

#### Dorsal Raphe Nucleus Projects to CDt

To our knowledge, this is the first report of CDt-projecting neurons in the DRN of monkeys and suggests DRN may provide serotoninergic inputs to CDt (Figure 12). Unlike previous studies (Parent et al., 1983; Smith and Parent, 1986), we found no DRN neurons labeled from the CDh injection site. These studies used large tracer injections covering much of CDh and putamen and found more putamen-projecting neurons than CDh-projecting neurons. This suggests parts of CDh may receive serotonergic DRN inputs, but the flexible-value CDh has few, if any, DRN inputs. Since DRN neurons encode expected and received reward values (Nakamura, 2013), they may contribute to the stable value coding of the CDt pathway.

#### Parasubiculum Projects to CDt

Within the parasubiculum, we found many CDt-projecting neurons. To our knowledge, this is the first report of projections from the parasubiculum to the caudate in primates. Two previous rodent studies (Groenewegen et al., 1987; Köhler, 1990) found projections from the subiculum to the caudate-putamen complex. Since the parasubiculum is implicated in spatial encoding (Taube, 1995; Hargreaves et al., 2007; Boccara et al., 2010), it is possible the parasubiculum sends information to CDt about specific spatial locations consistently associated with certain values, i.e., stable-value locations.

#### Technical Considerations

As with all tracer studies, damaged axons of passage near the injection site may have taken up tracer, especially given the numerous electrode penetrations needed to functionally identify each injection site. Uptake by axons of passage may have led to false anterograde and retrograde labeling, including false double-labeling of neurons. However, it is unlikely that this possible uptake heavily affected our results for several reasons. First, we only found anterograde labeling from the CTB555 injection site in the GPe and substantia nigra pars reticulata (SNr; Kim et al., 2014, 2017), both regions known to receive inputs from the caudate. Second, both monkeys had very similar results, which is unlikely if there was substantial uptake by axons of passage. Third, all of the injection sites had minimal leakage into the white matter compared to the amount of tracer within the caudate.

Another possibility is that the tracer leaked into the STC above the CDt injection site and was retrogradely transported to nearby layer 6 neurons in the STC and dorsal ITC. However, such cortico-cortical connections are unlikely to originate selectively from layer 6 (Usrey and Fitzpatrick, 1996; Wiser and Callaway, 1996; Zhang and Deschênes, 1997; Briggs and Callaway, 2001).

Another limitation of this study is the small injection sites compared to the total volume of the CDh and CDt. Our injection sites were localized to the areas with the most flexible- or stable-coding neurons of the CDh and CDt respectively, but the tracer did not cover all of the CDh and CDt that respectively encoded flexible and stable value. This current study captured many of the differences between the inputs to the CDh and CDt, but may still have missed differences between these two areas of the caudate.

## Conclusion and Future Questions

Overall, we found anatomical evidence for distinct pathways encoding flexible and stable values. Many cortical and subcortical regions contained CDh-projecting and CDt-projecting neurons in anatomically segregated subpopulations. One significant example of this was in STC and ITC, where cortical layer 5 contained CDh-projecting neurons and layer 6 contained CDt-projecting neurons. The functional differences between these layers are unknown, but investigations into this layer difference may reveal important information about laminar organization. Another significant example of this anatomical segregation was in the claustrum. Rostral-dorsal claustrum contained CDh-projecting neurons while caudal-ventral claustrum contained CDt-projecting neurons. Although claustrum’s function remains unknown, this anatomical finding may guide future functional studies. This tracer study raises many new questions about the functional roles of each of the cortical and subcortical inputs to the CDh and CDt in the flexible and stable value pathways. As these areas are further explored, they may give us more insight into how value memories are created, stored and recalled.

## Author Contributions

HFK performed recordings and injections. AG, WSG, HFK, MGC and KMW performed histology analysis. WSG analyzed the data with input from OH. WSG and OH wrote the article with input from other authors.

## Conflict of Interest Statement

The authors declare that the research was conducted in the absence of any commercial or financial relationships that could be construed as a potential conflict of interest.

## Abbreviations

AAA: Anterior amygdaloid area
ACC: Anterior cingulate cortex
BLA: Basolateral amygdala
CD: Caudate nucleus
CDh: Caudate head
CDt: Caudate tail
CL: Central lateral nucleus
CM: Centromedian nucleus
DRN: Dorsal raphe nucleus
EC: Entorhinal cortex
FC: Frontal cortex
FEF: Frontal eye field
ITC: Inferior temporal cortex
MD: Mediodorsal nucleus
LD: Lateral dorsal nucleus
LIP: Lateral intraparietal cortex
OFC: Orbitofrontal cortex
PC: Parietal cortex
PCC: Posterior cingulate cortex
PF: Parafascicular nucleus
PFC: Prefrontal cortex
PHC: Parahippocampal cortex
PMC: Premotor cortex
SNc: Substantia nigra pars compacta
SNr: Substantia nigra pars reticulata
STC: Superior temporal cortex
STN: Subthalamic nucleus
VA: Ventral anterior nucleus
VL: Ventral lateral nucleus.
